# *gsp* Mutation Is Not a Molecular Biomarker of Long-Term Response to First-Generation Somatostatin Receptor Ligands in Acromegaly

**DOI:** 10.3390/cancers13194857

**Published:** 2021-09-28

**Authors:** Luiz Eduardo Wildemberg, Daniel Henriques, Paula C. L. Elias, Carlos Henrique de A. Lima, Nina R. de Castro Musolino, Aline Helen Silva Camacho, Olivia Faria, Debora Nazato, Julio Abucham, Lucio Vilar, Jose Italo Mota, Martha Katherine P. Huayllas, Leila Chimelli, Margaret de Castro, Leandro Kasuki, Mônica R. Gadelha

**Affiliations:** 1 Endocrine Unit and Neuroendocrinology Research Center, Medical School and Hospital Universitário Clementino Fraga Filho, Universidade Federal do Rio de Janeiro, Rio de Janeiro 21941-617, Brazil; lwildemberg@gmail.com (L.E.W.); henriques.danielg@gmail.com (D.H.); oliviajfaria@gmail.com (O.F.); lkasuki@yahoo.com (L.K.); 2 Neuroendocrine Unit, Instituto Estadual do Cérebro Paulo Niemeyer, Secretaria Estadual de Saúde, Rio de Janeiro 20231-092, Brazil; 3 Division of Endocrinology, Department of Internal Medicine, Ribeirao Preto Medical School, Universidade de São Paulo, Ribeirão Preto 14049-900, Brazil; lamparelli@hotmail.com (P.C.L.E.); marga.castro50@gmail.com (M.d.C.); 4 Neuropathology and Molecular Genetics Laboratory, Instituto Estadual do Cérebro Paulo Niemeyer, Secretaria Estadual de Saúde, Rio de Janeiro 20231-092, Brazil; chazeredo@gmail.com (C.H.d.A.L.); alinehcamacho@gmail.com (A.H.S.C.); leila.chimelli@gmail.com (L.C.); 5 Neuroendocrine Unit, Division of Functional Neurosurgery, Hospital das Clínicas da Universidade de São Paulo, São Paulo 05403-000, Brazil; ninamusolino@gmail.com; 6 Neuroendocrine Unit, Division of Endocrinology and Metabolism, Escola Paulista de Medicina, Universidade Federal de São Paulo (Unifesp), São Paulo 04023-062, Brazil; debora.nazato@gmail.com (D.N.); julioabucham@uol.com.br (J.A.); 7 Division of Endocrinology, Hospital das Clínicas da Universidade Federal de Pernambuco, Recife 50670-901, Brazil; lvilarf@gmail.com; 8 Endocrinology and Metabolism Unit, Hospital Geral de Fortaleza, Secretaria Estadual de Saúde, Fortaleza 60150-160, Brazil; joseitalomota@hotmail.com; 9 Neuroendocrinology and Neurosurgery Unit, Hospital Brigadeiro, São Paulo 01401-000, Brazil; marthaka@uol.com.br

**Keywords:** somatostatin receptor ligands, *gsp* mutation, acromegaly, somatotropinoma

## Abstract

**Simple Summary:**

Acromegaly treatment consists of surgical, medical, and radiation therapy. First-generation somatostatin receptor ligands are the mainstay of medical therapy, with approximately 40% disease control rate. Several parameters have been evaluated as predictors of response to these drugs, including mutations in the stimulatory G-protein α subunit (*gsp* mutation), which is still controversial. In this study, we aimed to evaluate in a large series of patients whether *gsp* mutation predicts long-term response to medical treatment and to characterize the *gsp* mutated population. The ability to predict response to medical therapy would help to choose a therapy that presents higher odds of controlling the disease, which ultimately would reduce treatment costs and disease morbi-mortality.

**Abstract:**

Background: It is still controversial if activating mutations in the stimulatory G-protein α subunit (*gsp* mutation) are a biomarker of response to first generation somatostatin receptor ligands (fg-SRL) treatment in acromegaly. Thus, we aimed to evaluate whether *gsp* mutation predicts long-term response to fg-SRL treatment and to characterize the phenotype of patients harboring *gsp* mutations. Methods: *GNAS1* sequencing was performed by Sanger. SST2 and SST5 were analyzed by immunohistochemistry (IHC) and real-time RT-PCR. The cytokeratin granulation pattern was evaluated by IHC. Biochemical control was defined as GH < 1.0 ng/mL and normal age-adjusted IGF-I levels. Results: *gsp* mutation was found in 54 out of 136 patients evaluated. Biochemical control with fg-SRL treatment was similar in *gsp*+ and *gsp*- patients (37% vs. 25%, *p* = 0.219). Tumors harboring *gsp* mutation were smaller (*p* = 0.035) and had a lower chance of invading cavernous sinuses (*p* = 0.001). SST5 protein (*p* = 0.047) and mRNA (*p* = 0.013) expression levels were higher in wild-type tumors. Conclusions: In this largest series available in the literature, we concluded that *gsp* is not a molecular biomarker of response to fg-SRL treatment in acromegaly. However, the importance of its negative association with cavernous sinus invasion and SST5 expression needs to be further investigated.

## 1. Introduction

Acromegaly is a disease caused by growth hormone (GH) hypersecretion, leading to increased production and secretion of insulin-like growth factor type I (IGF-I) due, in most cases, to a GH secreting pituitary adenoma (somatotropinoma). First-line treatment is surgical resection, generally transsphenoidal surgery (TSS) [1]. However, surgical remission rates vary from 42 to 57%, depending on the experience of the institution [2,3]. The remaining patients ought to be submitted to a diverse treatment, which may include a second surgery, medical treatment, or radiotherapy [1]. First-generation somatostatin receptor ligands (fg-SRLs), namely, octreotide, and lanreotide, are the standard first-line medical treatment options for the majority of patients, with biochemical control rates varying from 19 to 60% [4].

The stimulatory G-protein α subunit, encoded by the *GNAS1* gene, is associated with growth hormone releasing hormone receptor signaling by the cAMP pathway, which is an important target of SRLs [5]. *GNAS1* activating mutations (*gsp* mutations) are found in approximately 40% of sporadic somatotropinomas [6], commonly found in codons 201 and 227, and are thought to be associated with smaller, densely granulated somatotropinomas that present higher GH secretion and higher somatostatin receptor subtype 2 (SST2) expression; however, this phenotype has not been clearly defined [7,8,9,10,11]. Another characteristic of patients harboring *gsp*+ tumors is a putative better response to fg-SRLs [11]. This issue has been studied for a long time but is still a matter of debate, since there is no consensus on whether it can predict response to fg-SRL or not [11,12,13]. A recent meta-analysis showed that *gsp*+ patients present a higher GH reduction during the octreotide suppression test (OST) [6]. However, studies concerning long-term response to fg-SRL treatment have presented conflicting results [11].

Based on data reported to date, the present study aims to evaluate whether *gsp* mutation predicts long-term response to fg-SRLs and to better characterize the phenotype of patients harboring *gsp* mutation.

## 2. Patients and Methods

### 2.1. Study Design

This is a retrospective longitudinal multicenter study, including seven Brazilian pituitary disease reference centers.

### 2.2. Patients

Patients with a confirmed diagnosis of sporadic acromegaly who underwent surgical treatment and had either fresh frozen or formalin-fixed paraffin embedded (FFPE) tissue available for analysis were included. SST2 and SST5 were evaluated exclusively in patients not medically treated prior to surgery.

### 2.3. Data Collection

Demographic, laboratory (GH and IGF-I levels), and imaging data at diagnosis (maximum tumor diameter and cavernous sinus invasion) and pre- and post-fg-SRLs treatment (at least six months on the highest approved fg-SRL dose) laboratory data were collected. Biochemical control was defined as the achievement of GH levels < 1.0 ng/mL and normal age adjusted IGF-I levels [1]. GH and IGF-I reduction after treatment was also evaluated. IGF-I was expressed as times the upper limit of normal (xULN). Cavernous sinus invasion was evaluated according to the modified Knosp–Steiner criteria [14]. Tumors classified as Knosp 3 or higher were considered invasive, whereas tumors classified as Knosp 2 or lower were considered noninvasive.

### 2.4. DNA and RNA Extraction

DNA and RNA were extracted from frozen tumor fragments and DNA from FFPE tissues using the AllPrep™ DNA/RNA/miRNA Kit (Qiagen, Hilden, Germany, Cat. No. 80224) and ReliaPrep™ FFPE gDNA Miniprep (Promega, Wisconsin, USA, Cat. No. A2352), according to the manufacturers’ protocols. At the end of the process, DNA and RNA were eluted in the provided buffer and stored at −80 °C until use.

For DNA extraction from FFPE tissues, six sections with 5-µm height and 1 cm^2^ were obtained, and effective deparaffinization with molecular biology-grade mineral oil was performed before DNA extraction. Incubation conditions to reverse crosslinking without the need for overnight digestion, xylene, or other hazardous or volatile solvents followed the manufacturer’s instructions.

### 2.5. DNA Sequencing

Fragments of *GNAS1* gene containing codons 201 and 227 were amplified by polymerase chain reaction (PCR) in a 25-μL reaction mixture with 2.5 µL of 10 x PCR buffer, 0.3 μg of DNA sample, 50 mM MgCl_2_, 10 mM dNTP mix, 10 μM of each primer and Platinum™ Taq DNA Polymerase (Invitrogen™, Waltham, MA USA) and nuclease-free water. Reactions were amplified at an initial denaturation temperature of 94 °C for 5 min, followed by 35 cycles of 94 °C for 45 s, 57.5 °C for 45 s, and 72 °C for 30 s. A final extension at 72 °C for 7 min was carried out to allow complete extension of amplified fragments. The amplified products were then visualized on a 2% agarose gel stained with SYBR™ Safe DNA Gel Stain (Invitrogen™, Waltham, MA USA).

PCR products were purified from unincorporated nucleotides and primers using ExoSAP-IT™ PCR Product Cleanup Reagent (Affymetrix™, Sant Clara, CA, USA). Direct sequencing of PCR products was performed in a 3130XL Genetic Analyzer automatic sequencer (Applied Biosystems, Life Technologies™, Waltham, MA, USA) and analyzed using Benchiling software (https://benchling.com/, accessed on 9 February 2021), CLUSTAL W (www.ebi.ac.uk/clustalw, accessed on 9 February 2021) and BioEdit 7.2.5 (http://www.mbio.ncsu.edu/BioEdit/bioedit.html, accessed on 9 February 2021)

### 2.6. qPCR

Quantitative real-time PCR was performed using the same primers and method as previously described to analyze the number of copies of *SST2* and *SST5* mRNA [15].

### 2.7. Immunohistochemistry

Immunohistochemistry was performed as previously described [16]. Rabbit monoclonal antibodies directed against SST2 (UMB-1, 1:5000, Abcam, Cambridge, UK, Cat. No. ab134152), SST5 (UMB-4, 1:2000, Abcam, Cambridge, UK, Cat. No. ab109495) and cytokeratin (CAM5.2) (1:10,000, Cell Marque, Rocklin, CA, USA, Cat. No. 452M-95) were used.

SST2 and SST5 were evaluated using the immunoreactive score (IRS), as previously described, and high expression was defined as an IRS > 5 [15]. For the cytokeratin pattern, tumors were classified as sparsely granulated (SG), densely granulated (DG), or intermediate (which were grouped with DG tumors) as previously described [17]. The samples were analyzed by two observers, and discordant results were submitted to an evaluation by a third observer.

### 2.8. Statistical Analysis

Results are expressed as median (min–max). Categorical variables were compared with Fisher’s exact test or a chi-squared test, as appropriate. Numerical variables were compared with a Mann–Whitney test. A *p*-value < 0.05 was considered significant.

## 3. Results

### 3.1. Patient and Sample Characteristics

One hundred thirty-six patients were included in this study; 63 (46%) of them were men, and median age at diagnosis was 43 years old (17–69). Median GH and IGF-I levels at diagnosis were 44.1 ng/mL (1.1–611) and 3.9 xULN (1.3–11.4), respectively. Out of 111 patients, 5 (5%) had microadenomas. Of these, data with respect to diameter were available in 69 patients, and the median maximum diameter was 20 mm (5–54). A cavernous sinus invasion was evaluated in 54 patients, including 23 (43%) invasive and 31 (57%) noninvasive tumors. Eighty-one (60%) patients were treated with fg-SRLs, and 24 (30%) of them were biochemically controlled.

**Figure 1:**
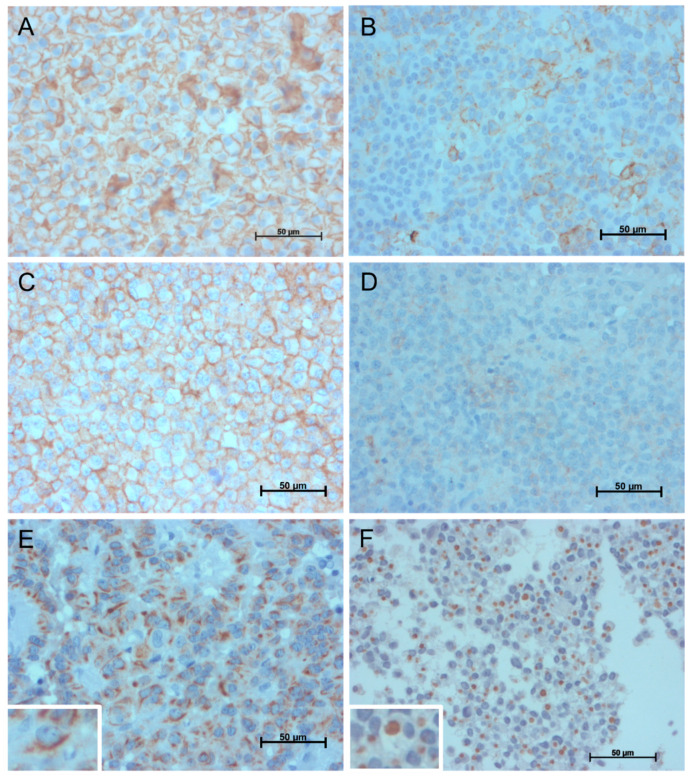
Representative images of immunohistochemical expression patterns of somatostatin receptors subtype 2 and 5 and cytokeratin. (**A**) micrograph showing membrane SST2 immunostaining in 100% of cells with high intensity (IRS score 12–high); (**B**) SST2 immunostaining in approximately 20% of cells with moderate intentisty (IRS 4–low); (**C**) SST5 immunostaining in 100% of cells with high intensity (IRS score 12–high); (**D**) SST5 immunostaining in less than 10% of cells with low intensity (IRS 1–low); (**E**) densely granulated tumor—inset shows a magnified view of the perinuclear staining; and (**F**) sparsely granulated tumor—inset shows a magnified view of the dot-like staining.

Cytokeratin granulation pattern was evaluated in 101 tumors. Forty (40%) were sparsely granulated, and 61 (60%) were densely granulated. SST2 and SST5 protein and mRNA expression were evaluated in 106 and 59 tumors, respectively. High SST2 expression was found in 79 (75%) tumors, and low expression was found in 27 (25%) tumors. Median *SST2* mRNA expression was 597 copies (12–3690), and it was significantly associated with protein expression (*p* < 0.001). High SST5 expression was demonstrated in 59 (56%) patients, whereas the remaining 47 (44%) had low expression. Median *SST5* mRNA expression was 83 copies (0.3–1318), and it was also associated with protein expression (*p* = 0.002). Examples of SST2, SST5, and granulation patterns are presented in Figure 1.

### 3.2. gsp Mutation

**Figure 2:**
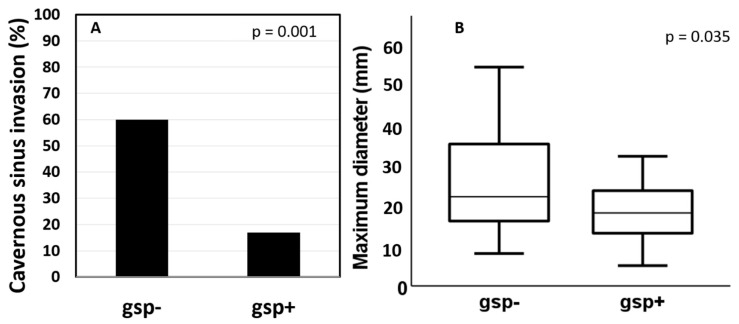
Association between *gsp* mutation and cavernous sinus invasion (**A**) and tumor maximum diameter (**B**). One outlier from the *gsp*+ group was excluded from the graph for better visualization: 42 mm.

**Figure 3:**
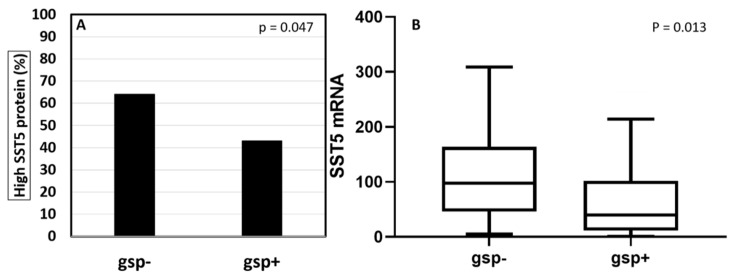
*gsp* mutation and SST5 protein (**A**) and mRNA (**B**) expression. Three outliers from the *gsp*- group (666, 1075, and 1318 copies) and one from the *gsp*+ group (214 copies were excluded from the graph for better visualization).

Among the 136 tumors analyzed, 54 (40%) harbored a *gsp* mutation. Forty-nine (91%) mutations were located at codon 201, and five (9%) mutations were located at codon 227. There was no correlation of tumor *gsp* status with sex (*p* = 0.858), age (*p* = 0.413), GH (*p* = 0.868), or IGF-I (*p* = 0.736) levels at diagnosis or with granulation pattern (*p* = 0.148). Frequency of macroadenomas was similar between *gsp-* and *gsp*+ tumors, but these tumors were smaller (18 mm vs. 22 mm; *p* = 0.035) and had a lower chance of cavernous sinus invasion (17% vs. 60%, *p* = 0.001) (Figure 2). No association was found between tumor *gsp* status and either SST2 protein or mRNA expression (*p* = 0.257 and 0.305, respectively). On the other hand, high SST5 protein expression was less frequent in *gsp+* tumors than in *gsp-* tumors (43% vs. 64%, *p* = 0.047), and *SST5* mRNA expression was lower in *gsp+* tumors (40 vs. 102, *p* = 0.013) (Figure 3). Table 1 summarizes these findings.

### 3.3. Predictors of Response to fg-SRL Treatment

Overall, biochemical control was found in 24 (30%) of patients. It was found in 11/30 (37%) patients with *gsp*+ tumors and in 13/51 (25%) patients with *gsp*- tumors (*p* = 0.219). Additionally, GH and IGF-I reduction after fg-SRL treatment were similar between patients with *gsp+* and *gsp-* tumors (*p* = 0.382 and 0.682, respectively).

On the other hand, SST2 protein and mRNA expressions were positively associated with biochemical control (*p* = 0.021 and 0.031, respectively). Association was also found with a granulation pattern (*p* = 0.037), but not with SST5 protein or mRNA expression (*p* = 0.894 and 0.399, respectively).

## 4. Discussion

Precision medicine has been increasingly used for disease management in different medical specialties, particularly oncology [18]. In acromegaly, prediction of response to SRL treatment has been the focus of several studies [19,20,21,22]. Numerous clinical, biochemical, immuno-histopathological, molecular, and imaging parameters have been studied with this purpose, including *gsp* mutation [23]. The present study is the largest series evaluating *gsp* status with respect to long-term biochemical response to SRLs published so far, including 136 patients, in which *gsp* mutation was found in 40% of cases. This high prevalence underscores the need to establish its significance in relation to the management of disease following surgical failure. Therefore, we evaluated its association with biochemical control in 81 patients, which was not found.

Attempts to correlate *gsp* mutation with clinical response to SRLs date from the 1990s, when Yang et al. [12] first described this association. They found that none of the five poor responders in an octreotide suppression test (OST) were *gsp*+, while three out of five good responders were *gsp*+. Since then, several studies have evaluated *gsp* mutations and their correlation with both acute and long-term responses to SRLs [6,7,8,9,10,13,24,25,26,27,28]. Recently, a meta-analysis encompassing 310 patients found 40% *gsp*+ tumors, similar to our results [6]. In this meta-analysis, patients harboring *gsp*+ tumors showed a greater GH level reduction during OST. It is important to emphasize that significant acute GH suppression does not necessarily correspond to a sustained long-term response [29]. If we take into consideration only studies evaluating long-term biochemical control, earlier reports showed a correlation with *gsp* mutation status [13,26], but these findings have not been confirmed in more recent studies [7,9,24,28]. Our data reinforce these later studies, particularly the study of Fougner et al. [24], who evaluated 38 patients and found no correlation of *gsp* mutation and GH or IGF-I reduction during OST or long-term fg-SRL treatment. There may be a concern that population heterogeneity could play a role in the differences found among studies. However, our study included patients from all over country, which presents a mixed population. Therefore, we consider that it is unlikely that these results may have been influenced by differences in the population, although it cannot be excluded that some genetic background has been underrepresented.

The phenotype of patients harboring *gsp* mutations is still controversial in the literature. We did not find any difference between patients with wild-type and *gsp* mutated tumors with respect to sex, age, GH, and IGF-I levels at diagnosis. However, tumors harboring *gsp* mutations are thought to be smaller, more frequently densely granulated, and more secretory [30]. Indeed, we found *gsp* mutated tumors to be smaller and less frequently invade cavernous sinuses, even when only macroadenomas were considered (data not shown). Of note, such invasion, characterized by the Knosp criteria, had never been evaluated in comparison to *gsp* mutation. Freda et al. [28] evaluated dura mater or bone invasion and found no difference between wild-type or *gsp*-mutated tumors, despite the latter being slightly smaller. Kim et al. [31] also did not find a difference in invasiveness between wild-type and *gsp*-mutated tumors classified according to the Hardy and Vezina grading system. Again, *gsp*-mutated tumors were significantly smaller.

With respect to granulation pattern, similar to Fougner et al. [24], we did not find a difference in the frequency of sparsely or densely granulated tumors between *gsp+* and *gsp-* tumors. It is important to note that ours and Fougner’s series are the largest described series of somatotropinomas.

A hypothesis that could favor a better response of *gsp*+ tumors to SRLs is the demonstration that SST may be differentially expressed between *gsp*- and *gsp*+ tumors, in particular, with a higher expression of *SST2* mRNA levels in the latter tumors [7,8,9]. However, these data have not been confirmed by other authors [13,32,33]. In our study, we did not find a significant correlation between *gsp*+ tumors and SST2 protein or mRNA levels.

In contrast, we did find that wild-type tumors express higher SST5 levels, both at the protein and mRNA levels. A previous finding of higher *SST5* mRNA levels in *gsp*- tumors had been described with borderline significance (*p* = 0.06) by our group [9]. The present series, aggregating a large number of patients from many Brazilian centers, allowed us to confirm the higher SST5 protein and gene expression, by two different very sensitive methods. In addition, we also confirmed a high correlation between *SST5* mRNA and protein, as previously demonstrated [34]. The clinical importance of the different SST2 and SST5 expression in the presence or absence of *gsp* mutation has not yet been investigated. Since SST5 expression has been shown to predict response to pasireotide in patients who are resistant to fg-SRLs [35], studies evaluating *gsp* mutations and the response to pasireotide treatment should be further examined in acromegaly.

We confirmed previous findings that showed association between SST2 expression and granulation pattern with response to fg-SRL [11,15,36,37]. Several other biomarkers have been shown to be associated with medical or surgical therapy response, but none with optimal predictive power [16,38,39,40,41]. Thus, there is still a need for better biomarkers or a combination of them. We recently developed a machine learning based prediction model, including clinical (age and sex), biochemical (GH and IGF-I levels), and immunohistochemical (SST2 and SST5 expression and cytokeratin granulation pattern) features, that predicted response to fg-SRL with an accuracy of 86.3%, positive predictive value of 83.3% and negative predictive value of 87.5% [42]. Another recent study evaluated clinical, biochemical, and imaging parameters, and found that age was negatively correlated with IGF-I reduction, whereas IGF-I levels at diagnosis and tumor T2 hypointensity at MRI were positively correlated [43].

## 5. Conclusions

The present study confirmed the frequency of *gsp+* mutations in approximately 40% of sporadic somatotropinomas. It also reinforced that, whereas *gsp+* tumors were significantly smaller than *gsp-* tumors, the phenotype did not differ with respect to sex, age, GH, and IGF-I levels at diagnosis or the association with biochemical control. This is the largest series evaluating *gsp* mutation status with respect to long-term medical treatment with fg-SRLs, and we concluded that the *gsp*+ mutation cannot be used as a molecular biomarker of long-term response to fg-SRLs. However, the higher expression of SST5 in *gsp+* tumors raises an important question regarding *gsp* mutation and the response to pasireotide treatment in acromegaly.

## Figures and Tables

**Table 1:** Comparison of demographic, laboratory, imaging, molecular, histopathological, and treatment response to first-generation somatostatin receptor ligands data between *gsp+ and gsp-* tumors.

	*gsp*+	*gsp*-	*p*-Value
Frequency–*n* (%)	54 (40%)	82 (60%)	NA
Sex (Male)–*n* (%)	24 (45%)	38 (46%)	0.858
Age (years)	45 (23–68)	43 (17–69)	0.413
GH at diagnosis (ng/mL)	46 (7–611)	42 (1.1–491)	0.868
IGF-I at diagnosis (xULN)	3.8 (1.3–10.2)	3.9 (1.3–11.4)	0.736
Diameter (mm)	18 (5–42)	22 (8–54)	0.035
Macroadenoma–*n* (%)	44 (96%)	62 (95%)	0.660
Cavernous sinus invasion–*n* (%)	4 (17%)	19 (60%)	0.001
*SST2* mRNA (copy number)	657 (69–1597)	415 (17–3690)	0.305
*SST5* mRNA (copy number)	40 (0.3–239)	102 (4.7–1318)	0.013
High SST2 IRS–*n* (%)	35 (85%)	44 (70%)	0.257
High SST5 IRS–*n* (%)	18 (43%)	41 (64%)	0.047
Sparsely granulated–*n* (%)	28 (70%)	33 (55%)	0.148
Biochemical control (%)–*n* = 81	11 (37%)	13 (25%)	0.219
GH reduction * (%)–*n*= 81	52 (−19–93)	44 (−41–88)	0.382
IGF-I reduction * (%)–*n*=81	47 (−4–83)	35 (−8–90)	0.682

*n*: number of individuals; NA: not applicable; GH: growth hormone; IGF-I: insulin-like growth factor type I; xULN: times the upper limit of normal; SST2: somatostatin receptor subtype 2; SST5: somatostatin receptor subtype 5; IRS: immunoreactivity score. * percentage of GH or IGF-I reduction with treatment.

## Data Availability

The data presented in this study are available on request from the corresponding author. The data are not publicly available due to privacy limitations.
